# Club Cell protein: a candidate diagnostic biomarker of Pseudomonas aeruginosa nosocomial pneumonia

**DOI:** 10.1186/cc13408

**Published:** 2014-03-17

**Authors:** V Moroz, A Kuzovlev, S Polovnikov

**Affiliations:** 1 V.A. Negovsky Scientific Research Insitute of General Reanimatology, RAMS, Moscow, Russia; 2 N.N. Burdenko Main Military Hospital, Moscow, Russia

## Introduction

Early etiological diagnosis of nosocomial pneumonia (NP) determines prompt targeted treatment. The aim of this study was to investigate the role of Club Cell protein (CCP) as a candidate diagnostic biomarker of Pseudomonas aeruginosa (PA) NP.

## Methods

The observational study in ICU ventilated septic patients with peritonitis (65%), pancreonecrosis (20%) and mediastinitis (15%) was performed in 2010 and 2013. Diagnosis of NP was made according to the standard clinical criteria. Associations of multiresistant Gram- negative bacteria were detected in sputum of all patients. PA was detected in 75% of patients. Plasma CCP was measured on the day of NP diagnosis (day 0) and days 3, 5 and 7 by the immunoenzyme essay (BioVendor, USA). Data were statistically analyzed by STATISTICA 7.0, ANOVA method, and presented as Me and 25 to 75 percentiles, ng/ml; *P *< 0.05 was considered significant. Areas under the receiver operating curves (ROC) were calculated.

## Results

**Figure 1:**
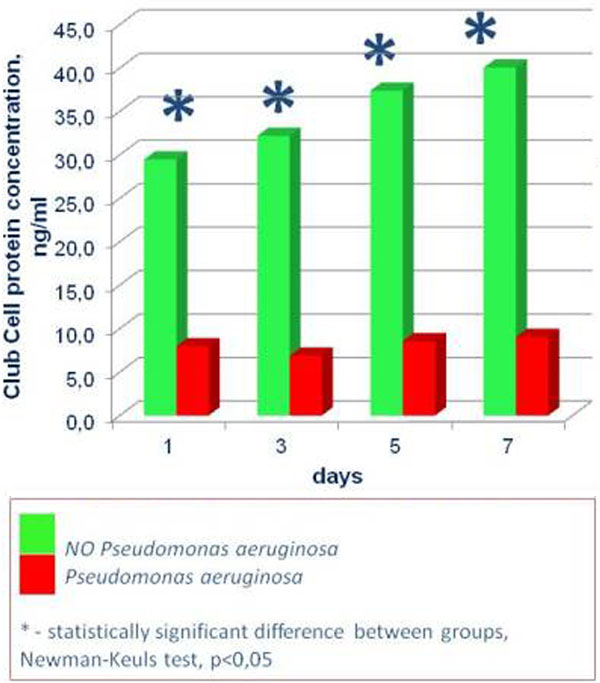
**Club Cell protein concentration in patients with Pseudomonas aeruginosa NP**.

**Figure 2:**
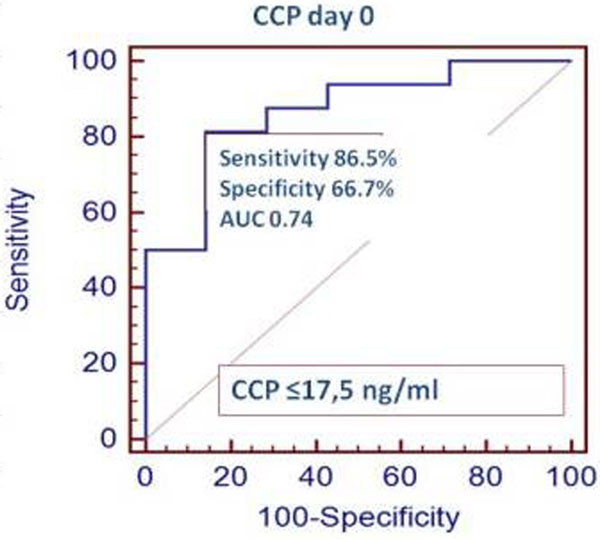
**ROC curve for Club Cell protein (day 0) for the diagnosis of Pseudomonas aeruginosa NP**.

Ninety patients (out of 350 screened) were enrolled in the study according to the inclusion/exclusion criteria. Patients were assigned to groups: NP (*n *= 50, 45 ± 4.3 years old, M/F 36/14) and noNP (*n *= 40, 48 ± 7.2 years old, M/F 30/10). Groups were comparable in APACHE II and CPIS scores. In patients with PA NP (*n *= 30), plasma CCP was significantly lower at all points than in the patients with no PA detected (*n *= 20; Figure 1). Plasma CCP on day 0 had a good capacity for the diagnosis of PA NP: CCP on day 0 ≤ 17.5 ng/ml yielded a sensitivity of 86.5% and specificity of 66.7% (AUC 0.74; 95% CI 0.630 to 0.829; *P *= 0.0001; Figure 2).

## Conclusion

Plasma CCP level ≤17.5 ng/ml is a sensitive and specific candidate diagnostic biomarker of PA NP.

